# Post-Genomics and Skin Inflammation

**DOI:** 10.1155/2010/364823

**Published:** 2010-09-19

**Authors:** Daniela Braconi, Giulia Bernardini, Annalisa Santucci

**Affiliations:** Dipartimento di Biologia Molecolare, Università degli Studi di Siena, via Fiorentina 1, 53100 Siena, Italy

## Abstract

Atopic dermatitis and psoriasis are two chronic skin inflammatory diseases that have so far received a greater attention within the scientific community through different post-genomic approaches; on the contrary, acne, which is undoubtedly one of the most common skin disorders involving inflammatory processes, seems to be still quite neglected under the post-genomic point of view. In this paper, we will review how post-genomic technologies have provided new fundamental tools for the analysis of these three conditions and we will cast light on their potential in addressing future research challenges.

## 1. Skin, Oxidative Stress, and Lipid Peroxidation

Skin represents the major interface between the body and the environment; consequently, it is chronically exposed to the harmful action of many chemical/physical toxicants. Besides forming a biological barrier to protect internal organs and systems from the external environment, skin can also symptomatically reflect internal diseases [1]. Both exogenously and endogenously generated compounds or their metabolites can directly or indirectly lead to the generation of a wide variety of reactive oxygen species (ROS), short-lived compounds that are normal by-products of aerobic metabolism. If generated in excess, ROS can quickly overwhelm the skin antioxidant defence systems. Such an imbalance in the production/inactivation of ROS is generally known as “oxidative stress” and is one of the causes for the damage of biological molecules (such as DNA, carbohydrates, lipids, and proteins) and the release of inflammatory cytokines [2, 3].

Lipid peroxidation (LPO) is basically deleterious because it leads to a diffuse spread of free radical reactions [4]. Membrane lipids, and mainly phospholipids containing polyunsaturated fatty acids (PUFAs) [5], are particularly prone to peroxidation because of their association within the cell membrane with nonenzymatic and enzymatic systems generating pro-oxidative free radical species. As a consequence, under various conditions and pathological states, an oxidative cascade may be generated that can, in turn, induce cytotoxicity and apoptosis and may have a significant role in inflammation enhancing the release of cytokines and modifying lipoproteins to proinflammatory forms [5, 6].

LPO products readily react with proteins, sugars, and DNA [7]. There are numerous reports indicating that end products of LPO, such as malondialdehyde (MDA), 4-hydroxy-2-nonenal (4-HNE), and 4-hydroxy-2-hexanal (4-HEE) can damage proteins by reacting with various amino acids both in vivo and in vitro [4]. At the same time, it has been shown over recent years that LPO products at low concentrations activate the cellular cytoprotective signaling pathways and increase antioxidant capacity [8]. The understanding of the effects of oxidative stress and LPO products in signal transduction represents the paradigm shift in the concept of oxidative stress, for which now different definitions exist. One of them is a notion that stress is a signal and oxidative stress is a signal which induces oxidative reaction and/or affects redox balance, resulting in either stimulation of defence capacity or induction of deleterious damage. As Selye, a pioneer in the field of biological stress, observed for diverse agents and coined the term [9], oxidative stress and LPO may turn to either positive stimulus, *eustress*, or deleterious insult, *distress* [7].

Oxidative stress has been already indicated as the underlying mechanism of various diseases, and compelling evidences have been produced to demonstrate a link between oxidative stress, especially LPO, and various inflammatory dermatologic conditions such as atopic dermatitis (AD) [10] and psoriasis [11]. Consistent with this notion, several groups have shown increased levels of LPO products in biological fluids and tissues from different pathological conditions. Nonetheless, whether LPO is the cause or the consequence of a disease condition is still an open question that needs to be addressed [7]. The fact that ROS, at moderate concentrations, can act as second messengers in the induction of biological responses and are specifically required as mediators/transduction molecules for signalling provides support to a correlation between oxidative stress and inflammation. On the other hand, an overproduction of ROS may completely imbalance the redox-homeostasis and irreversibly damage cell components, eventually leading to apoptosis. Oxidative stress, driving the production of lipid peroxides and their oxidation products, may thus be critical for the development of skin inflammatory diseases because an increased LPO may evoke immune responses, cause an excessive cytokine release, activate gene expression, affect cell proliferation, and induce apoptosis. Strengthening this hypothesis, it has recently been shown that the peroxisome proliferator-activated receptors (PPARs, whose natural ligands are PUFAs and their oxidation metabolites) may drive the development of psoriasis and acne [12].

## 2. Post-Genomics

The comprehension of skin diseases has advanced rapidly in the last several years, as dermatologic science is beginning to unravel the pathophysiology behind such diverse conditions. Though in vitro markers of LPO have been made available, their value can be limited by the fact that assays may lack sensitivity or require invasive methods to collect samples. Additionally, the choice among in vivo generated LPO products may pose relevant limits to the analysis, making the identification and quantification of these products more difficult. This is because such a determination in biological fluids and tissues takes into account and reflects the balance among formation, metabolism, clearance, and excretion of various compounds [4, 7].

The usefulness of biomarkers for the characterization of skin diseases is undoubted; nonetheless, their contribution could be read only in a very specific fashion. In fact, as seen, oxidative imbalances might affect many cellular functions, producing a complex scenario whose evaluation could be of greater interest and lead to a more detailed picture of a disease. As tools for systems biology are becoming increasingly available, it will be possible to analyse the generation, metabolism, and partitioning of LPO products together with their associated effects on cell molecular targets as well as to establish a correlation between alterations of specific proteins and a specific inflammatory skin disease. Efforts are already being made to apply post-genomic technologies aiming at a comprehensive characterization of skin inflammatory diseases through extensive mapping of cell components at the levels of the transcriptome and the proteome. This will likely ultimately lead to a picture of the signalling networks that mediate the biological effects of LPO [5].

In February 2001, the entire sequence of the human genome was first published [13, 14]. Once identified, the complete coding region of a gene is readily obtained. DNA microarrays, generating a high-throughput transcriptional profile of cells, can quantitatively analyze thousands of genes simultaneously and allow a global characterization of a specific molecular process. A growing body of evidences indicates that protein expression changes are important in skin diseases, and several large-scale expression profilings describing differential gene expression in skin diseases have been published. Nonetheless, they cannot provide information concerning the translational regulation of expression or posttranslational modifications (PTMs) of proteins, or qualitative and quantitative changes in the steady-state levels of proteins in biological systems. Investigators should then focus their attention on proteins, the results of gene expression, to determine how proteins might be responsible for the normal functioning cells. Only then the true role of a gene in the disease process can be understood. Proteomics is indeed the answer to these questions, as it can profile in a biological system the entire repertoire of proteins, including their isoforms and molecular species generated by PTMs (such as phosphorylation, glycosylation, oxidation, cleavage, and many others). The proteomic approach has been widely and extensively applied to the study of many human conditions whereas it is still in its infancy in cutaneous biology. However, the use of proteomics has the potential to elucidate which role specific proteins play in inflammatory skin manifestations and possibly speed up the identification of therapeutically relevant targets.

**Figure 1:**
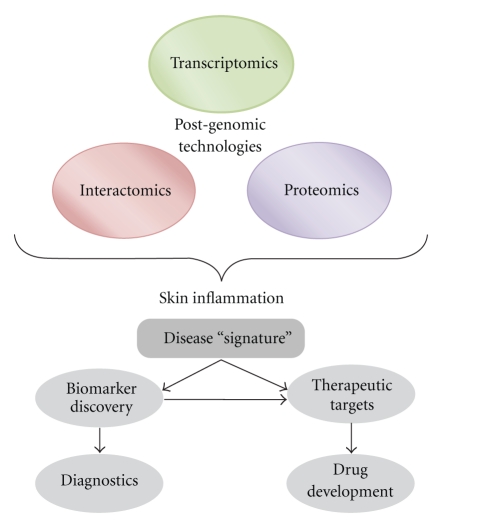
The integration of different post-genomic technologies for the study of skin inflammatory diseases.

Being proteins involved in complex protein networks, significant challenges must be faced in the study of protein functions and interaction maps. Functional proteomics and systems biology may hence provide new insights to gain a deeper understanding of cell systems and pathway properties also in skin diseases [15, 16]. Typically, proteins seek out other proteins, interaction partners, to complete their function(s). These partners may serve simply to hold proteins in particular orientation or involve structural modifications conferring new activities. By delineating these networks, scientists can gain an understanding of the role each individual protein plays in biochemical pathways, and thus a disease process. This knowledge can then be used for a focused approach to discover chemicals that might be effective in a disease state [17] (Figure 1).

Proteomics and transcriptomics have been applied to the study of several skin conditions, such as scleroderma, eczema, burn wounds, irritation induced by sodium lauryl sulphate, or cell responses to inflammatory interleukins. Several post-genomic studies have been dedicated so far to the study of two skin inflammatory diseases, that is, AD and psoriasis whereas acne, which is undoubtedly one of the most common skin conditions involving inflammatory processes, seems to be still quite neglected under the post-genomic point of view. In the present work, we will review how post-genomic technologies have been used and which is their potential in addressing future challenges able to provide vital insights on the pathogenic mechanisms of these three conditions.

## 3. Psoriasis

Psoriasis vulgaris is among the most prevalent chronic inflammatory skin diseases. Typically, the disease manifests as inflamed plaques prevalently in scalp, elbows, and knees. The plaques are characterized by erythema, induration, and silvery scaling. Hyperproliferative keratinocytes lack terminal differentiation, as seen by the formation of hyperparakeratotic stratum corneum [18]. In the typical picture of psoriatic skin, disregulated fatty acid metabolism, ROS generation, cytokine release, and LPO have all been reported [2].

A combination of genes is undoubtedly required to reveal the psoriasis phenotype after exposure to an environmental trigger, such as streptococcal pharyngitis or tonsillitis [19]. The elucidation of genes and gene products in psoriasis is of vital importance if targeted therapies are to be designed that may cure or possibly prevent the disease. Perhaps the most important consequence of identifying psoriasis genes is the ability to develop a transgenic animal model for psoriasis that will allow rapid and economic screening of potential therapeutic agents [20]. No such model exists, the nearest being an immunodeficient mouse xenografted with biopsies of human psoriasis [21].

Global comprehensive studies on gene expression in psoriasis have been carried out to elucidate the mechanisms of the disease. Particularly, the transcriptome (analysis of RNA expression profiling) has been investigated using different technological platforms: Gene Chips microarray [22–24], cDNA array [25], and Serial Analysis of Gene Expression (SAGE) [26]. These studies consistently found differential expression of genes related to cell regeneration, hyperkeratosis, metabolic functions, immune responses, and inflammation in lesional psoriatic skin and, altogether, provided a large number of novel gene markers. However, a detailed correlation between gene expression profiles of psoriatic skin and local and overall clinical parameters, as well as an analysis of the influence of the biopsy location on the gene expression profile in psoriatic skin, was not reported until the work by Quekenborn-Trinquet et al. [25]. In this work, using gene expression profiles of involved psoriatic skin through cDNA array and kinetically monitored reverse transcriptase-initiated polymerase chain reaction (kRT-PCR), authors compared gene expression profiles of plaque psoriasis at different anatomical sites for both symmetrical and asymmetrical diseases. Gene clustering failed to highlight any correlation with family history of psoriasis, age at onset, association with arthritis, and plaque location and type. Nonetheless, expression profile analysis highlighted distinct patterns according to both global (Psoriasis Area and Severity Index) and local (erythema, desquamation, and plaque elevation) clinical severity. Three novel psoriasis markers were identified in the work, namely SPRR2C (marker of abnormal keratinocyte differentiation), MX2 (transcription factor involved in mediating the proinflammatory effects of IFN-*γ*), and CYC1 (belonging to cytochrome-c family, indicates the high energetic request of highly proliferating epidermis of psoriatic lesions, also pointing out a transcriptional deregulation of proapoptotic factors) [25].

To identify differentially expressed genes which play causal roles in pathogenesis and maintenance of psoriasis, the BodyMap database combined with an RT-PCR-based amplified fragment length polymorphism (iAFLP) analysis was used by Itoh et al. 241 genes were found to be differentially expressed only in involved psoriatic skin, and seven genes showed high expression levels only in uninvolved psoriatic lesions. These seven genes, which were reported to be related to apoptosis or antiproliferation, might have causal roles in the pathophysiology of psoriasis [27].

A genomic approach was also undertaken by Lew et al. in order to discriminate the genomic signature of large plaque (Western) and small plaque (Asian) psoriasis [28]. However, authors led to the conclusions that these two manifestations share a similar genomic signature though a potential difference in IL-18 exists. Additionally, in the paper by Dezso et al. a computational methodology capable of predicting key regulatory genes and proteins in psoriasis is described. Using a dedicated algorithm and a global database of protein interactions, many biological targets of psoriasis were confirmed while several new ones were suggested [29].

Functional proteomics and systems biology are two emerging concepts that highlight the functional analysis of proteins and the networks they belong to. Applied to dermatological sciences, they can help researchers to get a better and clearer understanding of many skin diseases [15].

A pioneer proteomic approach to study psoriasis was carried out by Celis et al., who first established a 2D reference map for normal noncultured epidermal keratinocytes [30] and then revealed six low-molecular-weight proteins strongly upregulated in psoriatic skin that were proven to be partially released by cells [31]. These proteins included calgranulin A and B and cystatin A, while two of them were cloned, subsequently sequenced, and identified as “psoriasin” [32] and psoriasis-associated fatty acid-binding protein (PA-FABP) [33], pointing out a disregulated fatty acid metabolism in psoriatic cells. This observation was also in line with reports showing that the skin of psoriatic patients contains increased total lipids, phospholipids as well as free arachidonic acid and derived products.

The genetic background of psoriasis appears to be complex and whether different psoriasis phenotypes are associated with distinct genotypes is still unclear. Current research suggests that there is both clinical and genetic evidence for heterogeneity in psoriasis, but whether psoriasis actually consists of different diseases with distinct etiologic backgrounds has yet to be resolved. There is considerable overlap in phenotype expression and a single individual can display different psoriasis manifestations over time. Carlén et al. tried to identify differences between acute guttate and chronic plaque psoriasis at the protein level [34] using classical 2-DE plus Matrix-Assisted Laser Desorption/Ionization-Time Of Flight (MALDI-TOF) or Liquid Chromatography coupled with Mass Spectrometry (LC-MS/MS) for protein identification. The two phenotypes were clearly distinguishable and, interestingly, authors found a trend in which guttate psoriasis lesions clustered closer to eczema than to chronic plaque psoriasis lesions, indicating that the duration of the inflammatory reaction may affect clustering. This observation underscored the dynamic nature of skin inflammation in line with reports from AD, where the inflammatory and cytokine networks undergo significant changes over time [35, 36].

Intrinsic limitations characterize 2-DE analysis. First, 2-DE is not sensitive enough to detect proteins at pico-femto-molar levels, highly acidic or basic proteins, some membrane hydrophobic proteins, or very large (>250 kDa) and very small (<8 kDa) proteins. For all of these reasons, alternative approaches have been developed based on affinity-based protein purification strategies, variants of 2D-LC, LC coupled with capillary electrophoresis, and multidimensional LC in combination with MS [15]. For instance, Cowen et al. [37] undertook as MS analysis of serum samples from psoriatic patients (*serum proteomics*) to determine the proteomic signature of the disease. The rationale of the study relied on the fact that proteins associated with inflammatory processes in the skin are likely released into the extracellular space, entering the lymphatic vessels and, ultimately, the blood. Endothelial cells in skin also produce a variety of cytokines chemokines and adhesion molecules that are released directly into the circulation during inflammatory skin reactions. Both these processes may be expected to lead to quantitative as well as qualitative changes in the serum protein profile. Although facing the complexity of the serum proteome and the limitations of current protein technology, the work by Cowen et al. was an interesting pilot ‘proof of concept' study. This also because, due to the visibility and relatively easy access to affected tissues in cutaneous diseases, the combination of serum and tissue proteomic techniques might allow for potential correlation, validation, and reproducibility studies.

A similar approach, based on a combination of abundant protein depletion and multilectin affinity chromatography (M-LAC) combined with nanoflow liquid chromatography coupled with mass spectrometry (nano-LC-MS/MS), allowed Plavina et al. [38] to indicate galectin-3 binding protein (G3BP) as a protein marker for psoriasis.

Although the need for biomarker discovery for psoriasis is not pressing, because it is easily diagnosed from patients' symptoms, a better understanding of the disease pathogenesis is certainly needed to predict its severity, to identify new therapeutic agents, and for prognostic purposes. To address some of these issues, Plavina et al. [39] furthered their investigations on plasma from psoriatic patients using the approach described above to enrich glycoproteins together with a second method, based on ultracentrifugation and analysis of peptides by nano-LC-MS/MS with high accuracy Fourier transform, to analyze the low-molecular-weight (LMW) peptidome. The fruitful combination of proteomics and peptidomics allowed authors to detect increased concentrations of cytoskeletal proteins and their fragments in psoriatic plasma, suggesting disease-related cell leakage of these proteins and their increased proteolysis. Moreover, authors found increased concentrations of two proteins known to play a role in a genetic predisposition to psoriasis and in the differentiation of keratinocytes: calgranulins A and B. On this basis, it was hypothesized that the overproduction of calgranulins may alter calcium homeostasis in the epidermis and lead to altered relocalization of cytoskeleton components and their abnormal proteolysis, as well as to immune responses against circulating cytoskeletal proteins. Another striking finding was a decrease in fibrinogen fragments, suggesting a possible involvement in autoimmunity and inflammation and letting authors hypothesize a link between altered proteolysis of fibrinogen due to changes in its PTM and the development of psoriatic arthritis.

## 4. Atopic Dermatitis

AD is a chronic relapsing inflammatory skin disease frequently associated with skin infections and impaired skin barrier function [40]. The disease can seriously compromise the quality of life as it is characterized by the distribution of eczematous skin lesions. AD typically reveals lichenification, pruritic excoriations, and dry skin with a variety of pathophysiologic manifestations. The extrinsic type of AD (ADe) is associated with IgE-mediated sensitization and accounts for 70%–80% AD cases, while the intrinsic type (ADi) does not involve IgE and accounts for 20%–30% of AD cases. It is generally recognized that a combination of environmental factors such as allergens and bacterial infection, as well as genetic factors, is the principal inducers of the multiple immunologic and inflammatory responses seen in AD patients [41]. An impaired oxidative status has been already associated to AD, and systemic alterations in antioxidant patterns have been found in involved skin of AD patients as well as in uninvolved nonlesioned skin as an adaptive response to chronic inflammation of the epidermis [2].

Filament aggregating protein (filaggrin, FLG) is a key protein that plays an important role in the formation of the cornified cell envelope (CCE), which is critical for an effective skin barrier [42]. Genetic studies have shown that loss-of-function (null) mutations in the gene encoding FLG result in impaired skin barrier in AD [43–45]. Howell et al. demonstrated that *FLG* gene expression and FLG protein are decreased in the skin of patients with AD and indicated that this deficiency is caused, in part, by the overexpression of T_H_2 cytokines IL-4 and IL-13 that downregulate *FLG* expression during keratinocyte differentiation. This implies that many patients with AD acquire the FLG deficiency with subsequent barrier disruption as the result of the local inflammatory immune response [46]. However, *FLG* mutations alone do not provoke AD because the mutations can be observed only in a group of patients from certain ethnic groups (as many as 20% in all patients with AD and 50% in patients with severe AD) [47]. Similar mutations were also reported in approximately 9% of the European population without concomitant inflammation [43].

In 2009, Guttman-Yassky et al. [47] reported a comprehensive gene (mRNA) expression profile of AD and psoriasis by using normal skin as a reference. Although both the diseases share epidermal hyperplasia and regenerative epidermal growth, terminal differentiation was found to be accelerated in psoriasis but broadly suppressed or defective in AD. Authors found that psoriasis and AD can be correctly discriminated on the basis of differences in expression of terminal differentiation genes, much like disease distinctions made on the basis of immune-response phenotypes (“immune-signature” based on a set of 10 immune related genes).

Though the incidence of AD is rapidly increasing, a treatment offering quick relief of symptoms still remains to be developed. Various methodological approaches along with the new technologies can be fruitfully combined to this aim, especially DNA microarray and interactomic tools [41]. With DNA microarray, although obtaining a great number of disregulated genes in a disease, it is very difficult to choose the most important genes and it is hard to explain the meaning of encoded proteins. In this regard, interactomic tools may help find the core proteins among the detected candidate genes. Since the next step of functional studies is a time- and cost-consuming process, the number of target proteins must be limited; hence, to make the right choice, computational prediction on the basis of microarray results could be critical to piece together such a complete puzzle [48]. In light of this, Lü et al. [48] attempted to profile gene alterations of AD keratinocytes and corroborated microarray results with protein-protein interaction (PPI) mapping to predict the genes related with AD functioning as interaction hubs. In punch biopsies from ADi patients, they identified 25 predicted hub genes and two important candidate biomarker genes (*MMP1* and *MMP10*) significantly upregulated, both at the cellular (keratinocyte) level and in serum. In a large scale DNA chip array, the same group focused on ADe and used 205 candidate genes to carry out PPI prediction through bioinformatic analysis. The identified hub genes were mainly involved in five functional classes: ECM and cell adhesion, ATP/GTP binding, immune response, proteolytic enzymes, and structural genes [48].

Gene chip arrays were used in AD with various purposes, including:

- the identification of gene expression specific to active atopic skin lesions [49] such as the abnormal epidermal differentiation and defective defences found by Sugiura et al. as key abnormalities in AD [50];
- the identification of gene transcription changes associated with early AD inflammation as potential disease control targets [51];
- the pattern comparison between gene expression in AD *versus* psoriasis in an effort to identify potentially new gene targets that may be useful in the diagnosis and treatment of skin diseases [52];
- the understanding of why patients with AD, but not psoriasis, frequently suffer from serious skin infections [53].

Both proteomic and gene expression profilings of AD fibroblasts were carried out by Park et al. They investigated alterations in protein expression in primary cultured AD cells from ADe and ADi biopsy samples by classical 2-DE and MALDI-TOF [54] and additionally extended their analysis with the use of different pH gradients [55] to increase the possibility to detect protein markers. They also reported, notably, that PTMs of candidate proteins may be relevant in the development of the disease [54].

Then, Park et al. investigated the protein repertoire of primary cultured keratinocytes from AD samples and reported alteration of lipid metabolism-associated proteins within these cells, providing important clues to explain the presence of dry skin and pruritus in AD patients, as well as new insights into the identity of metabolic enzymes involved in AD pathogenesis [56]. In further works, Park et al. carried out a cDNA microarray analysis of fibroblasts from ADe and ADi where they suggested that human dermal fibroblasts may actively participate in AD disease [57] and proposed acetaldehyde dehydrogenase 1 (ALDH1) as a biomarker for AD disease using proteomic profiling of fibroblasts from ADi and ADe samples [58].

Howell et al. undertook a Differential In Gel Electrophoresis (DIGE) proteomic approach to investigate the global effects of IL-4 and IL-13 (which are well known to play a role in AD) on protein expression in keratinocytes. Notably, authors identified factors contributing to the significant barrier disruption and antimicrobial peptide (AMP) deficiency observed in the skin of AD patients and identified novel targets to enhance keratinocyte differentiation and skin innate immune responses in this common skin disease [59].

Glycoproteome is a major subproteome, since many clinical biomarkers and therapeutic targets are glycoproteins. In particular, the glycosylation patterns are influenced considerably by the physiological status [60, 61]. In their work, Kim et al. carried out a differential glycoprotein profiling of plasma from healthy donors and AD patients and identified several differentially expressed proteins suggested as potential biomarkers in the diagnosis of AD or in tracking its progress [62].

The goal of the recent pilot study by Broccardo et al. [63] was to develop an MS-based, noninvasive skin taping technique to study AD in infants and young children. In fact, though the disease affects mainly this age group, there are no skin-based studies on such a population because of the invasiveness of sampling biopsies. Besides confirming previous reports on unique proteins identified in AD samples, this proof-of-principle pilot study revealed also new potential biomarkers to investigate AD development and persistence.

## 5. Acne

Affecting millions of people worldwide, acne is the most common skin inflammatory condition. Although an incompletely understood pathogenesis, acne has been linked to multiple factors such as increased sebum production, inflammation of hair follicles and sebaceous glands, follicular hyperkeratinisation, and colonization by *Propionibacterium acnes* [78]. Nonetheless, only a minor number of works based on post-genomic technologies could be found on acne, highlighting the quite neglected nature of this disease.

The first comparative gene array expression profiling between inflammatory acne lesions and normal skin was carried out by Trivedi et al. in 2006 [79] and showed that many of the genes whose expression is increased in acne lesions are involved in inflammatory processes, the major genes including the matrix metalloproteinases *MMP-1* and *MMP-3*, as well as the pro-inflammatory cytokine *IL-8* and *CXCL-2*. This finding, together with the induction of several AMPs (e.g., granulysin) and proapoptotic enzymes (e.g., granzyme B), supported the correlation between inflammatory mediators and the extent to which *P. acnes* induce the expression of the AMP *β*-defensin (now known as DEFB-4) and IL-8 [80]. Critical questions remain, however, about the nature of the initiating events in the development of acne lesions. It is likely that gene expression profiles of inflammatory process in the skin are quite similar and that many of the changes observed in inflammatory lesions are secondary to as yet unidentified primary pathogenic events. The challenge lies ahead in identifying these primary events in acne as well as in other inflammatory diseases [79].

Unlike humans, most animals produce little or no triglycerides in hair follicles to harbour *P. acnes*, a fact that has encumbered the development of in vivo models. Although genetic mutant mice with acne-like skins have been used for screening antiacne drugs, the mice generally have deficits in immune system making them inappropriate to generate antibodies for developing acne vaccines. Nakatsuji et al. undertook a bioengineering approach to create a humanized acne microenvironment where proteomics using isotope-coded protein label (ICPL) coupled to nano-LC-MS analysis was carried out to investigate in vivo interactions among *P. acnes*, human sebocytes, and host immune cells. Thirteen proteins were identified, including secreted proteins and cell matrix proteins derived from mouse, human cells, or *P. acnes*, indicating the coexistence of protein repertoires from different origins. Altogether, these results may provide new pharmacological targets for the treatment of acne and a valuable model for drug screening and vaccine development [81].

## 6. Challenges and Future Directions: Redox-Proteomics and Immunoproteomics

**Figure 2:**
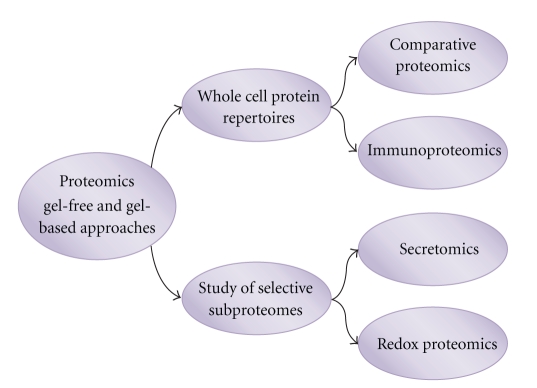
Approaches and strategy for the analysis of protein repertoires.

Proteomics of skin inflammatory diseases is still in its infancy and much remains to be done. For instance, the combined use of 2-DE and Western Blot (*immunoproteomics*), the investigation of cell secretomes (*secretomics*), and the discovery of oxidative PTMs of proteins have so far received little attention in this field of research (Figure 2).

PTMs play key roles in many important cellular functions by influencing protein subcellular localization, protein-protein interactions, and cellular biological activities. Altogether, PTMs finely orchestrate regulatory processes as well as cellular responses upon specific stress conditions. If irreversible, PTMs might be associated with permanent protein loss of function and may lead to the elimination or to the accumulation of the damaged proteins. If reversible, particularly at the cysteine residues, PTMs may have the dual role of protection from irreversible oxidation and modulation of protein function (redox regulation) [82].

Relying on both gel-based and gel-free approaches, several techniques are available and offer tremendous opportunities to analyze PTMs, helping in the elucidation of pathophysiological mechanisms in diseased or stressed cells. Since oxidative stress may induce several modification on proteins, the term “redox-proteomics” was coined to describe the multiple applications of proteomics for the discovery of oxidative PTMs. Nonetheless, an accurate identification of protein PTMs in relation to skin inflammatory diseases is still lacking.

In Table 1, some of the most commonly investigated oxidatively induced protein PTMs are reported, together with the reagents and techniques for their detection. Among these PTMs, the introduction of carbonyl groups has probably received the greatest attention within the scientific community. Carbonylation of proteins is an irreversible, easy detectable, and nonenzymatic PTM often accompanied by protein loss of function. For these reasons, it has been investigated widely in human diseases and it is universally accepted as a reliable indicator of oxidative stress [83–85]. The levels of protein carbonyls in a sample and especially the identification of target proteins through 1-DE or 2-DE and WB have been reported in several organisms and under many pathological conditions, providing evidences for evolutionarily conserved carbonylation patterns [86] and suggesting a role for the regulation of fundamental cellular events, including apoptosis [87–89].

Despite the insights provided by these studies, only recently the identification of the exact amino acid site and type of carbonyl modification has emerged. This information is necessary for a deeper understanding of the oxidative mechanisms leading to the protein modification as well as for providing information for the assessment of the functional effects that modification of specific amino acid sites may have on proteins. Therefore, the continuous evolution of MS techniques might drive researchers towards this important goal [90]. As an example, gel-free methods using enrichment of carbonylated peptides followed by direct LC-MS/MS analysis have been developed [72]. Interest has also been dedicated to the identification of modifications induced by 4-HNE relying on LC-ESI-MS/MS [70], as well as tryptic digestion, avidin column enrichment of the modified peptides, and MS/MS analysis following N′-aminooxymethylcarbonylhydro-D-biotin labeling [71].

Sulfur-containing amino acids are among the best susceptible targets of PTMs during oxidative stress [91]. Proteins thiol groups play a fundamental role in numerous cell processes, and their redox state is involved in both structure and function of many receptors, enzymes, and transcription factors. Thiol oxidation often leads to alterations in conformation and catalytic activity of proteins [92]; nonetheless, these events do not necessarily have to be seen as damaging. If, on the one hand, thiol oxidation could be a “random” event, on the other, it can be part of finely tuned processes that protect proteins from irreversible oxidation or activate specific functions related to stress response. The availability of different labelling reagents and electrophoretic techniques offers researchers important tools to unravel the importance of thiol oxidation in skin inflammatory diseases.

ROS may also be involved in skin immune manifestations [2, 3] and LPO has been already linked to autoimmune diseases. Reaction between MDA and endogenous proteins might lead to their covalent modifications, resulting in structural changes in the proteins, which might act as neoantigens inducing an autoimmune response. In fact, significantly increased anti-MDA epitope(s) autoantibodies were found in patients with systemic diseases, such as scleroderma, supporting the hypothesis that aldehydic degradation products of LPO may contribute to the autoimmune pathogenesis [93]. Additionally, some aldehyde modifications have been demonstrated to evoke strong antibody reactivity also against nonmodified proteins [94]. More generally, free radicals and aldehydes produced by chronic inflammation can induce a number of alterations, including gene mutations and PTMs of key proteins, eventually leading to disruption of cellular processes. Driving the production of *α*,*β*-unsaturated fatty aldehydes including MDA and 4-HNE, LPO can ultimately result in the production of protein adducts and in the elicitation of specific autoantibody formation [95, 96].

 For all of these reasons, understanding how proteins are actually affected by oxidative stress and inflammation is vital. Proteomics and immunoproteomics, in particular, might help the elucidation of disease aetiology. In the case of skin idiopathic diseases for which an immune response may be hypothesized, the role of this methodological approach can be two-fold. On the one hand, if the immune response is elicited by a specific pathogen, the relationship between the pathogen and the disease may be obtained and the antigens (molecular mimicry) concurring to the disease manifestation may be identified if WB analysis is carried out using patients' serum probed against the pathogen protein repertoire [97]. On the other hand, proteomics and immunoproteomics may help the identification of protein PTMs inducing conformational alterations evoking immune/autoimmune responses.

## Figures and Tables

**Table 1:** Oxidative PTMs of proteins and techniques for their study.

oxidative PTM	description	
	gel-based approaches + MS protein identification	gel-free approaches
3-nitroTyrosine	WB detection with *α*-nitroTyr antibodies [64–68]	

4-HNE-adducts	WB detection with *α*-4-HNE antibodies [64, 69]	LC-ESI-MS/MS, as well as tryptic digestion, avidin column enrichment and MS/MS analysis following N′-aminooxymethylcarbonylhydro-D-biotin labeling [70, 71]

Carbonylation	derivatization of protein carbonyls with 2,4-dinitrophenylhydrazine (DNPH) and WB detection of the resulting 2,4-dinitrophenylhydrazones (DNP) with *α*-DNP antibodies; alternatively, biotin-hydrazide tagging and avidin-FITC staining [64, 66, 68, 69, 72, 73]	enrichment strategies with the hydrazide biotin-streptavidin methodology or Girard's P reagent, coupled with LC-MS/MS and MALDI-MS/MS [66, 69–71]

Glycosylation	(i) lectin chemistry (due to a different specificity for certain carbohydrates, the identification of subclasses is allowed) [65]	(i) lectin affinity chromatography to isolate glycosylated proteins from complex mixtures prior to MS/MS [74]
	(ii) periodate/Schiff's base chemistry to generate a general stain toward glycoproteins (Pro-Q Emerald staining) [65]	(ii) chemical trapping of *N*-glycosylated peptide prior to LC-MS/MS [74]
		(iii) chemoenzymatic or tagging *via* substrate strategies for O-glycolsylated peptided [74]
		(iv) COFRADIC [74]

Oxidation of -SH groups	various techniques to reveal specific -SH PTMs [66, 68, 73, 75]	
	(i) oxidation, lack of labelling with specific reagents, such as biotinylated iodoacetamide, and WB detection with streptavidin	(i) isolation of cysteinyl peptides by biotinylation of Cys residues and affinity isolation (ICAT) [74]
	(ii) S-glutathionylation, metabolic labelling of the intracellular glutathione pool with ^35^S-cysteine while inhibiting protein synthesis plus nonreducing electrophoresis, and autoradiography	(ii) S-nytrosilation, modified biotin switch method coupled with affinity isolation [74]
	(iii) S-nytrosilation, biotin switch method	(iii) COFRADIC [74]
	(iv) formation of disulphide bridges, diagonal 2-DE	
	(v) sulfinic/sulfonic acid: detection through MS after standard trypsin digestion	

Phosphorylation	(i) in gel protein staining with specific dies (Pro-Q Diamond) [65, 76]	(i) isolation of phosphopeptides by immobilized ion chromatography (IMAC) [74]
	(ii) WB with antibodies towards specific phosphorylated amino acids	(ii) segregation of phosphopeptides by strong cation exchange chromatography or titanium dioxide [74]
		(iii) various chemical reactions aiming at modifying the phopshorylated peptides prior to MS/MS [74]
		(iv) COFRADIC [74]

multiple PTMs	SEMSA during LC-ESI-MS/MS [77]	

## List of Abbreviations

2-DE: Two Dimensional Electrophoresis
4-HEE: 4-hydroxy-2-hexanal
4-HNE: 4-hydroxy-2-nonenal
AD: Atopic Dermatitis
AMP: AntiMicrobial Peptide
COFRADIC: COmbined FRActional DIagonal Chromatography
DIGE: Differential In Gel Electrophoresis
ESI: ElectroSpray Ionization
ICAT: Iisotope-Coded Affinity Technology
LC: Liquid Chromatography
LPO: Lipid PerOxidation
MALDI-TOF: Matrix-Assisted Laser Desorption/Ionization-Time Of Flight
MDA: MalonDiAldehyde
MS: Mass Spectrometry
PTMs: PostTranslational Modifications
PUFA: PolyUnsaturated Fatty Acids
ROS: Reactive Oxygen Species
SEMSA: Selectively Excluded Mass Spectrometry Analysis
WB: Western Blot.
